# Supplementary material for: DMRforPairs: identifying Differentially Methylated Regions between unique samples using array based methylation profiles
Source: BMC Bioinformatics. 2014 May 15;15:141. doi: 10.1186/1471-2105-15-141 (PMC4046028; doi:10.1186/1471-2105-15-141)
Supplement: Additional file 2 — DMRforPairs output for the comparison of A431-MCF7 and NA17018-NA17105. Please start from the HTML files in each folder. Available via the BMC Bioinformatics website. [file 1471-2105-15-141-S2.zip › 1394847754114233_MOESM2_ESM/A431_MCF7/figures/13012.pdf]

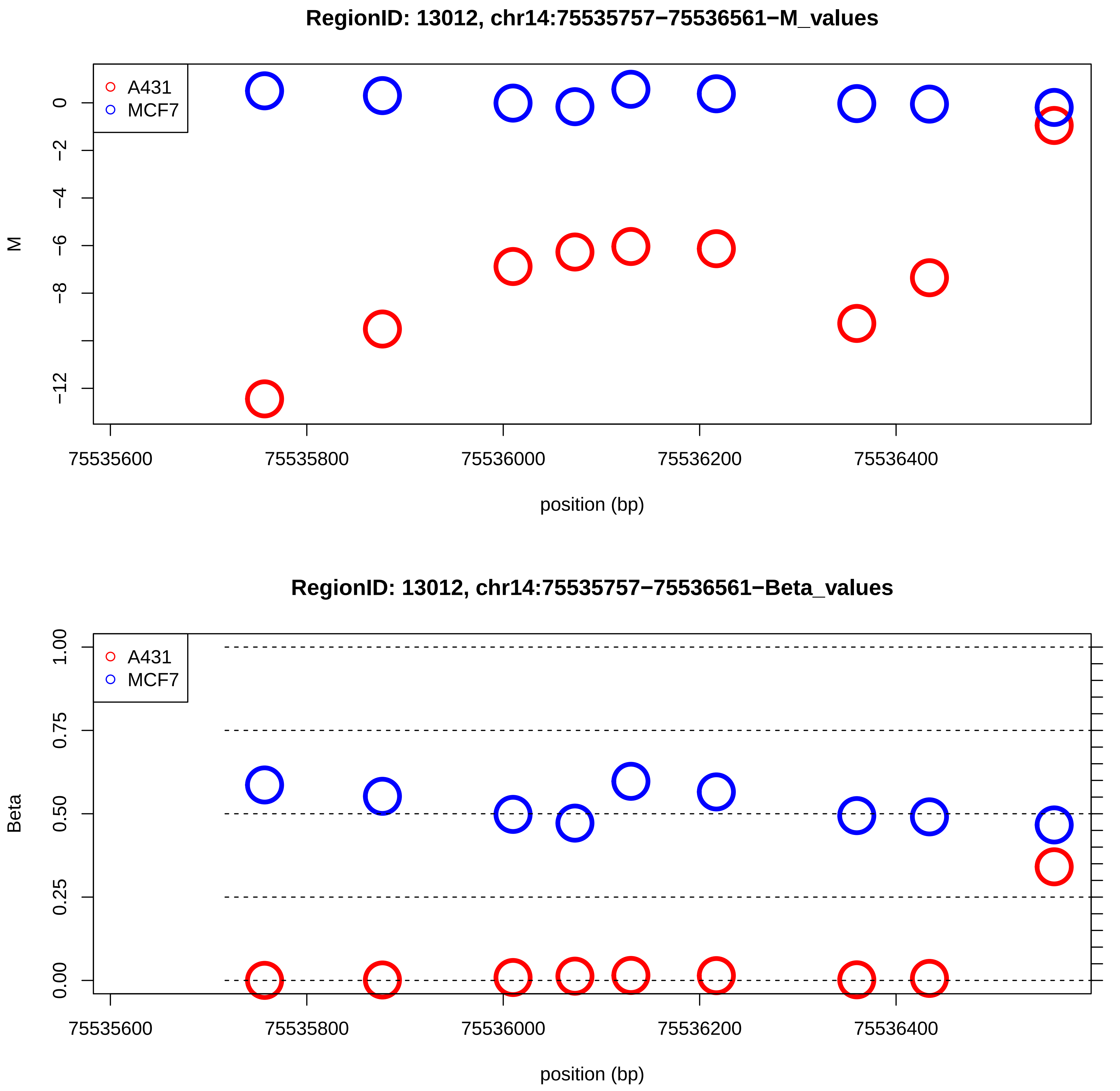
RegionID: 13012, chr14:75535757−75536561−M_values
A431
MCF7
M
0
−2
−4
−6
−8
−12
75535600
75535800
75536000
75536200
75536400
position (bp)
RegionID: 13012, chr14:75535757−75536561−Beta_values
A431
MCF7
Beta
0.00
0.25
0.50
0.75
1.00
75535600
75535800
75536000
75536200
75536400
position (bp)

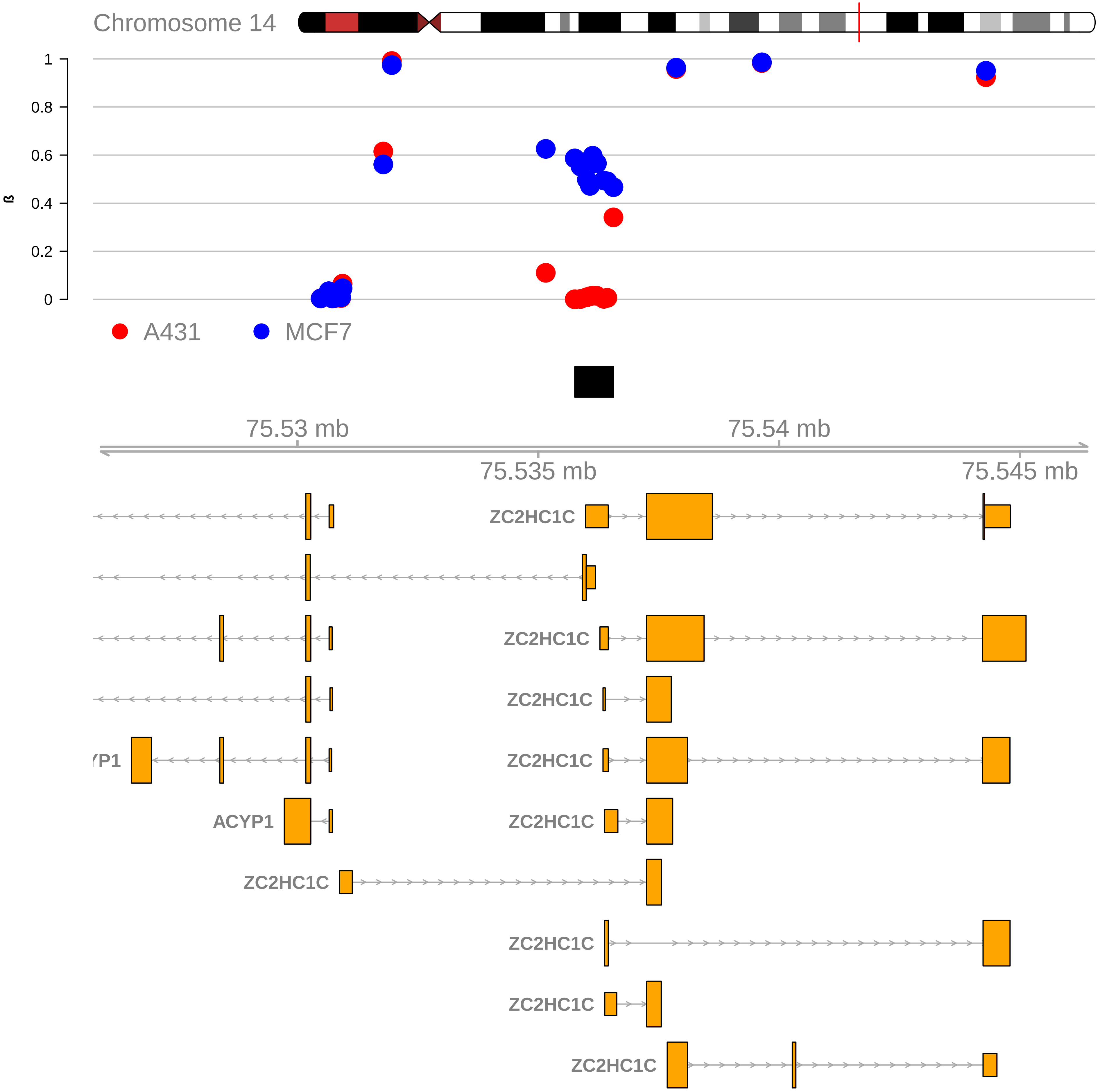

Chromosome 14
1
0.8
0.6
ß
0.4
0.2
0
A431
MCF7
75.53 mb
75.54 mb
75.535 mb
75.545 mb
ZC2HC1C
ZC2HC1C
ZC2HC1C
/P1
ZC2HC1C
ACYP1
ZC2HC1C
ZC2HC1C
ZC2HC1C
ZC2HC1C
ZC2HC1C
